# Quantification of Surgical Workflow during Robotic Proctectomy

**DOI:** 10.21203/rs.3.rs-3462719/v1

**Published:** 2023-10-20

**Authors:** Mishal Gillani, Manali Rupji, Courtney Devin, Lilia Purvis, Terrah Paul Olson, Anthony Jarc, Mallory Shields, Yuan Liu, Seth Rosen

**Affiliations:** Emory University School of Medicine; Winship Cancer Institute; Emory University School of Medicine; Intuitive Surgical; Emory University School of Medicine; Intuitive Surgical; Intuitive Surgical; Emory University; Emory University School of Medicine

**Keywords:** robotic, proctectomy, objective performance indicator, annotation, surgical workflow

## Abstract

**Aim:**

Assessments of surgical workflow offer insight regarding procedure variability, case complexity and surgeon proficiency. We utilize an objective method to evaluate step-by-step workflow and step transitions during robotic proctectomy (RP).

**Methods:**

We annotated 31 RPs using a procedure-specific annotation card. Using Spearman’s correlation, we measured strength of association of *step time* and *step visit frequency* with console time (CT) and total operative time (TOT).

**Results:**

Across 31 RPs, a mean (± standard deviation) of 49.0 (± 20.3) steps occurred per procedure. Mean CT and TOT were 213 (± 90) and 283 (± 108) minutes. Posterior mesorectal dissection required most visits (8.7 ± 5.0), while anastomosis required most time (18.0 [± 8.5] minutes). Inferior mesenteric vein (IMV) ligation required least visits (1.0 ± 0.0) and lowest duration (0.9 [± 0.5] minutes). Strong correlations were seen with CT and step times for IMV dissection and ligation (ρ = 0.60 for both), lateral-to-medial splenic flexure mobilization (SFM) (ρ = 0.63), left rectal dissection (ρ = 0.64) and mesorectal division (ρ = 0.71). CT correlated strongly with medial-to-lateral and supracolic SFM visit frequency (ρ = 0.75 and ρ = 0.65). There were strong correlations with TOT and initial exposure time (ρ = 0.60), as well as visit frequency for medial-to-lateral (ρ = 0.67) and supracolic SFM (ρ = 0.65). Descending colon mobilization was *nodal*, rectal mobilization *convergent* and rectal transection *divergent*.

**Conclusion:**

This study correlates individual surgical steps with CT and TOT through standardized annotation. It provides an objective approach to quantify workflow.

## Introduction

Assessment of surgical workflow is critical for evaluating surgeon skill, case complexity and surgery variability. The scarcity of procedure-specific annotated data hinders quantitative analysis of surgical workflow, consequently limiting the ability to provide targeted feedback and objective insights [1]. Subjective operative reports are currently the primary source of information regarding surgical workflow. As part of an overall movement towards optimized surgical care, there is a need for more objective and standardized operative reporting.

Operative reports dictated by surgeons have been shown to lack accuracy and are subject to bias [2–5] . Using descriptors like “hostile abdomen,” “extensive scar tissue,” or “extremely difficult dissection” are inherently subjective. There is an ongoing effort towards more objective reporting of operative events during surgical procedures. For example, in colon and rectal cancer reporting, synoptic operative reports are now required by the American College of Surgeons and the National Accreditation Program for Rectal Cancer [6].

Currently, console time (CT) and total operative time (TOT) are commonly used as quality metrics to assess robotic surgeon proficiency, but these metrics do not accurately reflect other factors that influence surgical workflow. For example, CT and TOT may be affected by patient factors (obesity, prior radiation), variations in pathology (bulky tumors), surgical team expertise, or involvement of surgical trainees (dual-console procedures). There is therefore a need for more objective, surgeon-specific data pertaining to surgery workflow. Robotic surgery, in which computer hardware and software is situated between the patient and surgeon, allows a unique opportunity for recording and analyzing objective data reflective of surgical workflow during live surgery.

Machine learning-enabled metrics, *objective performance indicators (OPIs)*, can be captured from surgical video and robotic system data. OPIs include measurements of surgeon console actions (hand, wrist and foot movements, clutching, energy use, arm swaps) and patient cart activity (camera movements, instrument path length, travel time, velocity, acceleration, jerk), as well as other calculated indices (economy of motion, smoothness, workspace volume) [7–9]. A specific OPI, *step duration*, enables accurate time-stamping of critical steps within a given procedure and provides a tool to evaluate surgical workflow and its variability across cases. A prerequisite for step-specific analyses is the ability to annotate surgical videos so as to identify discrete, functionally relevant surgical steps. An annotation card can be used to label start and stop times for steps during videos of robotic surgery. It has been utilized by Ghodoussipor et al. for partial nephrectomy [10], Tousignant et al. for sleeve gastrectomy [11], and Metchik et al. for lobectomy [12], but no such work has been published for proctectomy. Through step segmentation, OPIs have enabled objective step-specific feedback to provide insights into surgical workflow, procedure complexity, surgeon skill and surgical outcomes [12–14]. Although there is no universally accepted annotation card for RP, we have identified 21 steps during RP, and have previously published regarding our annotation process [9].

In this study, we objectively describe step-specific workflow and transitions within RP. Additionally, we identify critical steps that highly correlate with CT and TOT, as well as steps more likely to be *convergent* (varied steps precede, but few follow), *divergent* (few steps precede, but varied follow), or *nodal* (varied steps precede and follow), providing novel insights into workflow variabilities of RP. This segmentation and objective characterization of surgical workflow will aid in future development of synoptic operative reporting.

## Methods

The protocol of this study was approved by the institutional review board (IRB) of Emory University (IRB #00111214).

### Data Collection and Step Annotation

31 RPs conducted at Emory Hospitals between 2020 and 2021 were included in this study. An Intuitive Data Recorder (Intuitive Surgical, Sunnyvale, CA) was utilized to capture endoscopic video synchronized to robotics system data. No changes to patient management, surgical decision-making or surgeon assignment were made due to IDR recording.

A procedure-specific annotation card was developed using a Delphi method. After reviewing other scorecards for assessing colon and rectal surgery, as well as pertinent literature, our group of six colorectal surgeons went through 6 Delphi rounds to create a final annotation card. The annotation card was trialed during video review of 4 RPs in an auditing phase, and modifications were made to improve consistency and accuracy. The final annotation card for this study was utilized by professional video annotators to determine start and stop times of individual RP steps.

The 21 individual steps of RP include inferior mesenteric artery dissection, descending and sigmoid colon mobilization, splenic flexure mobilization and posterior mesorectal dissection. To allow for a clear beginning and end to each step, we defined ‘start’ and ‘stop’ parameters. As an example, the start parameter of inferior mesenteric artery (IMA) dissection is “the first instrument interaction with the mesentery surrounding the IMA.” The stop parameter is an IMA that “is skeletonized and ready for transection.” Throughout the procedure, a surgeon may skip individual steps, perform them more than once, or revisit them multiple times.

### Step Correlation and Classification

We evaluated time spent on each step, number of times each step was visited, CT, TOT and step transitions. We classified specific steps based on the number of visits and their relationship to other steps. *Nodal* steps describe a step with at least 10 different steps preceding and 10 different steps following. *Divergent* steps were defined as a step with fewer than 10 different steps preceding and more than 10 different steps following, and *convergent* steps were defined as having more than 10 different steps preceding and fewer than 10 different steps following.

#### Statistical Analysis:

Statistical analyses were conducted using SAS Version 9.4. Categorical variables were presented with counts and percentages and the numeric variables with median and interquartile range (IQR) or mean and standard deviation (SD), as appropriate based on normality. Individual step time and step visit frequency were reported using mean and standard deviation, and their associations with CT and TOT were measured using Spearman’s correlation. Circos plots, widely used for visualizing genome data, were employed to visually represent step transitions [15]. To achieve this, we constructed a matrix in which rows denote the frequency of *divergent* steps, while columns represent the frequency of *convergent* steps. The Circos application was employed to transform this matrix into chord diagrams. These plots facilitate visual variation of large data in a relatively small space by placing steps in a circular layout and drawing unidirectional links (ribbons) that represent transitions between them.

## Results

### Patient demographics

Indications for 31 RPs included cancer (23), diverticulitis (3), polyp (2), rectal prolapse (1), inflammatory bowel disease (1) and endometriosis (1). Mean age of the cohort was 59.8 (SD: 12.5) years, with the majority (62.5%) being female. Average body mass index was 27.8 (SD: 7.2) kg/m^2^ and mean American Society of Anesthesiologist score was 2.5 (SD: 0.5). Patients were hospitalized for a median of 4.0 [IQR: 4.0 – 6.0] days. Post-operative complications included ileus or small bowel obstruction (16.1%), acute renal injury (6.5%), cardiac event (6.5%), bleeding (6.5%) and hypoxia (3.2%). According to the Clavien-Dindo (CD) classification, 9/12 (75.0%) of the complications were rated as CD I and II, and 3/12 (25.0%) as CD III.

### Step visits and step times

The mean number of step occurrences per procedure was 49.0 (± 20.3). Posterior mesorectal dissection and right lateral rectal dissection occurred during all procedures and required the most step visits, with a mean of 8.7 (± 5.0) and 7.6 (± 5.2) steps per procedure, respectively (Table 1). On the other extreme, inferior mesenteric vein (IMV) dissection and IMV ligation occurred in only 4 procedures, with a mean of (± 0.9) and 1.0 (± 0.0) step visits per procedure, respectively.

Mean CT and TOT across all analyzed cases were 213 (± 90) and 283 (± 108) minutes. The steps with the longest durations were colorectal anastomosis (18.0 [± 8.5] minutes), mesorectal division (15.7 [± 10.7] minutes) and posterior mesorectal dissection (13.4 [± 13.1] minutes). IMV ligation needed the shortest duration, with a mean of 0.9 [± 0.5] minutes.

### Step correlation with CT and TOT

To elucidate the surgical steps most predictive of operative time, we calculated correlations of individual steps with CT and TOT. The following showed strong correlations with CT and step duration: IMV dissection and ligation (r = 0.60 for both), lateral-to-medial splenic flexure mobilization (SFM) (r = 0.63), left lateral rectal dissection (r = 0.64), and mesorectal division (r = 0.71). CT also correlated strongly with medial-to-lateral and supracolic SFM visit frequency (r = 0.75 and r = 0.65). In regard to TOT, there was a strong correlation for initial exposure time (r = 0.60), as well as for medial-to-lateral SFM and supracolic SFM visit frequency (r = 0.67 and r = 0.65, respectively) (Table 1).

### Step transitions and Circos plots

To determine the spectrum of transitions, a subset of all the RP steps that met specific requirements were defined as nodal (varied steps precede and follow), *divergent* (few steps precede, but varied steps follow) or *convergent* (varied steps precede, but few follow). *Nodal* steps included medial-to-lateral and lateral-to-medial mobilization of the descending and sigmoid colon (Table 2). On the other hand, the medial-to-lateral mobilization of the rectum was identified as a *convergent* step (13 types of steps precede, 7 follow), and transection of the rectum (6 types precede and 12 follow) was classified as a *divergent* step.

**Figure 1:**
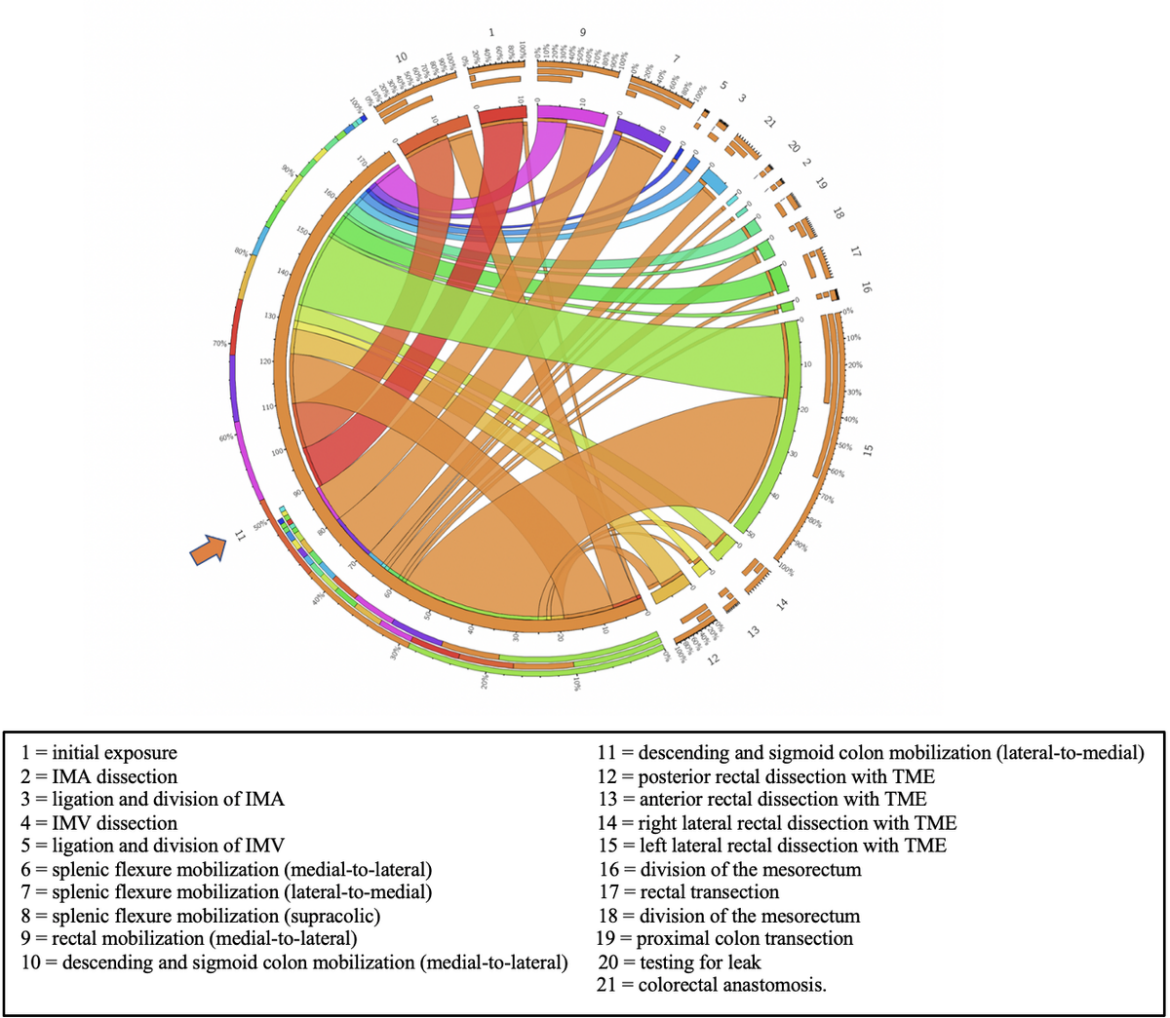
Lateral-to-medial descending and sigmoid colon mobilization (step 11, indicated by the arrow) is observed as a nodalstep across RP cases. Circos plot illustrates convergence and divergence of all steps with respect to step 11. It is predominantly preceded and followed by left lateral rectal dissection (step 15). Numbers correspond to the surgical steps as described.

**Figure 2:**
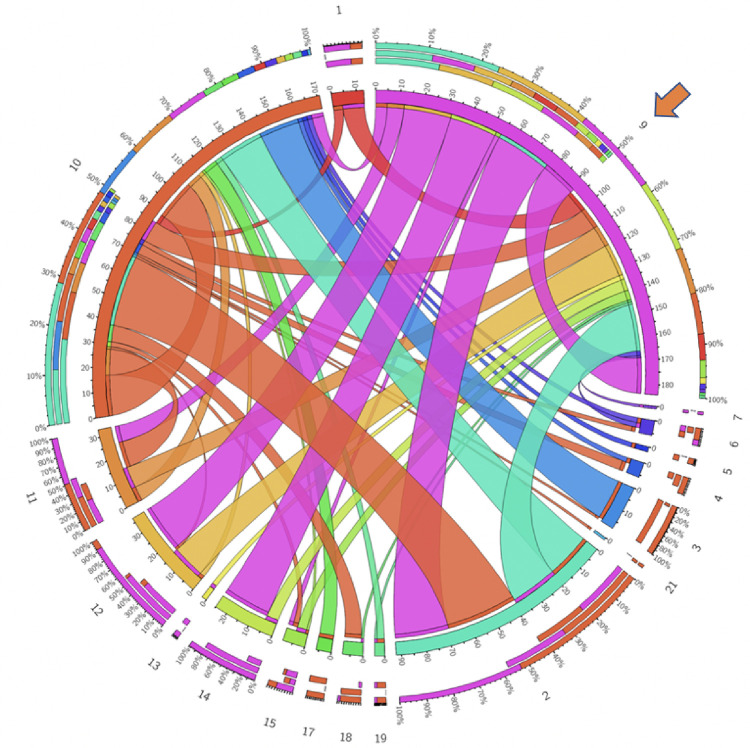
Medial-to-lateral mobilization of rectum (step 9, indicated by the arrow) is observed as a convergentstep across RP cases. Circos plot illustrates the convergence and divergence of all steps with respect to step 9. It is predominantly preceded by IMA dissection (step 2), and followed by IMA dissection (step 2) and posterior rectal dissection (step 12). Numbers correspond to the surgical steps as described in Figure 1.

**Figure 3:**
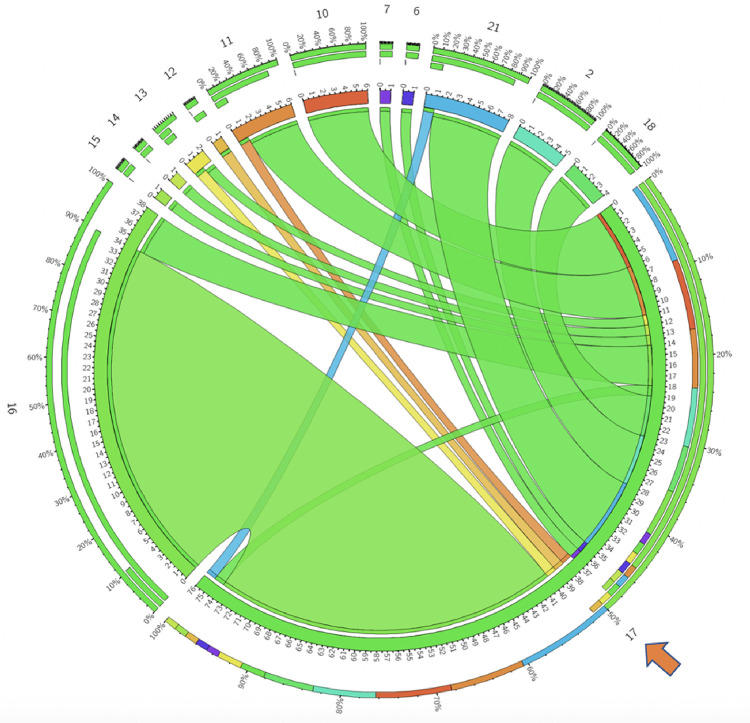
Rectal transection (step 17, indicated by the arrow) is observed as a divergent step across RP cases. Circos plot illustrates the convergence and divergence of all steps with respect to step 17. It is predominantly preceded by division of the mesorectum (step 16), and followed by medial-to-lateral descending and sigmoid colon mobilization (step 10) and colorectal anatomosis (step 21). Numbers correspond to the surgical steps as described in Figure 1.

While the nodal, convergent, and divergent step categories offer a broad framework for assessing the dynamics of RP, a Circos plot can offer a visual representation and frequencies of transitions from one step to another. Figure 1 shows the Circos plots for one of the *nodal* steps, while Figures 2 and 3 demonstrate *convergent* and *divergent* steps. In these plots, the outermost circle represents the entire dataset, and each subsequent inner circle represent subsets (steps). The ribbons represent step transitions and their thickness corresponds to the frequency of transitions, indicating the magnitude of step changes. If the ribbon originates from a color-filled area and transitions into a white space on another step label, it signifies a movement from the colored step to the white step. Ribbons entering and exiting the same step indicate frequent transitions within that specific step. The frequency and percentages for each step are represented on a scale surrounding the circos plots.

## Discussion

In this study, we quantitatively captured surgical workflow, including step transitions, thereby providing novel insights into the dynamics of RP. We also correlated two objective, scalable metrics (*step time* and *step visit frequency*) with more tradition metrics of proficiency (CT and TOT) [16, 17].

Utilizing the Delphi method, we developed a procedure-specific annotation card for RP, which allowed us to delineate each of the 21 steps of the operation. The annotation method has been used for other procedures across multiple specialties including laparoscopic colectomy, laparoscopic gastric bypass, sleeve gastrectomy, and transsphenoidal endoscopic pituitary resection, all with excellent consensus [18–20]. An annotation card was used to objectively define individual surgical steps, discover which require multiple visits, which require the most or least amount of time, and which significantly correlate with CT and TOT. Steps were defined as *nodal*, *convergent*, or *divergent* in order to understand the flow of operations and detect trends in steps as they relate to one another.

Rectal dissection in all four quadrants (anterior, posterior, right, left) required the highest amount of step visits, with posterior and right lateral dissections requiring significantly more visits than the others. As the majority of these procedures were for cancer, it is not surprising that these steps required many visits. Presumably, surgeons used meticulous attention and technique during these steps, with a focus on achieving excellent radial and distal margins. Regarding the laterality of rectal dissection, it is noteworthy that right lateral dissection visits were significantly higher than on the left side. This is likely due to camera placement in the right lower quadrant of the abdomen during RP, leading most surgeons to dissect right to left for more than half the dissection. Colorectal anastomosis, mesorectal dissection, and posterior rectal dissection required the longest mean times. Many of our anastomoses were performed intracorporeally, which will obviously require more console time. Mesorectal dissection requires precise use of a monopolar instrument and vessel sealer to achieve a linear, perpendicular distal margin, while avoiding bleeding or rectal wall injury. Posterior rectal dissection is a critical step during total mesorectal excision (TME). This step requires multiple camera and retraction adjustments, and careful, deliberate bimanual dissection to develop the golden plane behind the mesorectum, but anterior to the presacral nerves.

CT and TOT may not accurately reflect surgeon skill as there are intra-operative confounders, including surgical team proficiency and concurrent training (dual-console procedures), that may influence surgical times. Additionally, patient-related factors such as obesity, adhesions, history of radiation, or more advanced pathology, can influence CT and TOT [21, 22]. For these reasons, as well as others, we are exploring more precise and comprehensive ways of understanding surgical workflow and its effects on CT and TOT.

We discovered numerous strong correlations between step visit frequency and step time with both CT and TOT. Splenic flexure mobilization (both step frequency and step time) had strong correlation with CT and TOT. This makes sense, as complete splenic flexure mobilization was usually not performed, and when done was indicative of a more difficult reconstruction. Similarly, longer initial exposure time had a strong correlation with TOT. Longer initial exposure times were usually due to dense adhesions from prior surgery requiring extended adhesiolysis. Mesorectal dissection and left rectal dissection both had a strong correlation with CT. These steps require precise dissection and many adjustments to achieve and maintain exposure. They are more complicated in obese patients, those with bulky tumors, a narrow pelvis or prior radiation. Additionally, IMV dissection and ligation showed strong correlation to CT. IMV dissection and ligation, like splenic flexure mobilization, were not routinely performed and appear to have wider variation in step times.

Both nodal steps were related to dissection of the descending and sigmoid colon (medial-to-lateral and lateral-to-medial approaches), with greater than 10 different steps both preceding and following. Medial-to-lateral dissection of the rectum was a convergent step, with 13 different steps preceding (most commonly IMA dissection) and only 7 different steps following. Rectal transection was a divergent step, with 6 different steps preceding, and 12 different steps following. The most common procedure following rectal transection was colorectal anastomosis. The variation in step dynamics can be due to differences in patient anatomy, varied pathology, surgeon preference, or in response to operative events. For example, some surgeons started with lateral-to-medial dissection of the abdominal colon, followed by medial-to-lateral pelvic dissection and TME. Other surgeons began with medial-to-lateral pelvic dissection, followed by IMA dissection and ligation, then TME, and lastly medial-to-lateral abdominal colon mobilization. By precisely annotating these procedures, and correlating objectively delineated workflow with predictors (patient demographics, specific pathology, surgeon experience) and results (operative times, patient outcomes), we hope to more accurately understand what exactly occurs during these procedures, with more precise answers for when and why.

We will utilize our current findings to inform future research in the field of *surgical data science*, an emerging area of research focused on studying objective performance indicators during robotic surgery. By focusing on the most influential steps of a procedure, we hope to understand which OPIs (i.e., kinematic and event data) are most predictive of surgeon skill and case complexity. Subsequent studies will include a larger sample size, surgeons with different experience levels, and patients with varied diseases. This work may help define stages of learning curve across key steps of an operation. Specifically, we anticipate assigning percentage of individual step ownership by trainees, and tracking the progression of step ownership throughout a trainee’s curriculum. Ultimately, this data will be instrumental in identifying best training practices for new robotic surgeons.

Limitations to this study include a limited sample size from a single institution, the inclusion of both single and dual console cases, and the heterogenous nature of surgical indications. Even with these limitations, this study provides critical insights into surgical workflow and dynamics of RP. As such, future studies are needed to address these limitations and build upon these promising results.

## Conclusion

Through utilization of a procedure-specific annotation card for RP, our study identifies a novel approach to quantitatively define surgical workflow. We have identified *step time* and *step visit frequency* as useful OPIs to track surgical workflow, and found correlations with traditional metrics, CT and TOT. We found duration of splenic flexure mobilization, mesorectal dissection, left rectal dissection and IMV dissection are strong predictors of CT, while initial exposure time is a strong predictor of TOT. SFM step frequency was also a strong predictor of CT and TOT. Additionally, we identified mobilization of the descending and sigmoid colon, medial-to-lateral rectal dissection and rectal transection represent nodal, convergent, and divergent steps, respectively. These findings will be instrumental in informing future OPI research on critical aspects of robotic colorectal surgery. Our study provides an objective method to measure surgeons’ progression through cases, thereby serving as a foundation for targeted interventions aimed at shortening the learning curve and enhancing clinical outcomes.

## Figures and Tables

**Table 1:** Surgical step times and step visits, with correlations to total operative time and console time

	Step Time (min)				Step Visits			
Step Name	*Σ^1^*	*Mean (±SD)*	*r TOT*	*r CT*	*Σ* ^2^	*Mean (±SD)*	*r TOT*	*r CT*
Initial Exposure	235.11	9.04 ± 11.4	**0.60**	0.09	31	1.19 ± 0.49	−0.12	−0.03
IMA dissection	249.12	8.90 ± 5.4	0.12	−0.07	92	3.29 ± 1.88	0.17	0.37
Ligation & division of IMA	39.16	1.40 ± 0.9	−0.18	0.40	32	1.14 ± 0.45	0.34	0.32
IMV dissection	16.96	3.39 ± 2.7	0.42	**0.60**	8	1.60 ± 0.89	0.22	0.34
Ligation & division of IMV	5.55	0.92 ± 0.5	−0.14	**0.60**	6	1.00 ± 0.00	NA	NA
Splenic flexure mobilization (medial-to-lateral)	142.15	12.92 ± 9.5	0.49	0.58	44	4.00 ± 3.38	**0.67**	**0.75**
Splenic flexure mobilization (lateral-to-medial)	105.81	10.05 ± 7.7	0.59	**0.63**	34	2.27 ± 1.98	0.50	0.49
Splenic flexure mobilization (supracolic)	124.79	12.83 ± 13.6	0.57	0.57	19	2.71 ± 1.60	**0.65**	**0.65**
Rectal mobilization (medial-to-lateral)	232.28	8.01 ± 7.4	0.13	0.33	92	3.17 ± 2.41	−0.07	0.123
Descending & sigmoid colon mobilization (medial-to-lateral)	255.16	9.45 ± 8.4	0.31	0.41	86	3.19 ± 2.34	−0.07	0.15
Descending & sigmoid colon mobilization (lateral-to-medial)	305.56	10.19 ± 8.2	0.11	0.33	90	3.00 ± 1.74	0.14	0.25
Posterior rectal dissection	400.68	13.36 ± 13.1	0.57	0.35	261	8.70 ± 5.00	0.42	0.22
Anterior rectal dissection	164.71	7.16 ± 6.0	0.29	0.10	118	5.13 ± 3.31	0.43	0.23
Right lateral rectal dissection	123.34	4.36 ± 5.1	0.41	0.38	220	7.59 ± 5.18	0.30	0.19
Left lateral rectal dissection	177.95	5.93 ± 4.3	0.59	**0.64**	135	4.50 ± 3.13	**0.66**	0.56
Division of the mesorectum	454.68	15.68 ± 10.7	0.55	**0.71**	78	2.69 ± 1.75	0.34	0.41
Rectal transection	210.04	7.50 ± 5.8	0.40	0.49	39	1.39 ± 0.57	0.13	−0.07
Division of the mesocolon	222.05	8.54 ± 4.3	0.37	0.47	37	1.42 ± 0.50	0.04	0.14
Proximal colon transection	58.18	2.53 ± 2.0	0.36	0.37	25	1.09 ± 0.29	0.32	0.38
Testing for leak (optional)	53.05	2.79 ± 2.1	0.07	0.04	26	1.37 ± 0.96	0.42	0.50
Colorectal anastomosis	396.52	18.02 ± 8.5	0.16	0.19	45	2.05 ± 1.68	−0.11	−0.13

*Σ^1^* = cumulative time in minutes during 31 operations

*Σ^2^* = cumulative number of step visits during 31 operations

min = minutes, SD = standard deviation, *r =* Spearman’s rank correlation coefficient, TOT = total operative time, CT = console time, IMA = inferior mesenteric artery, IMV = inferior mesenteric vein

**Table 2:** Nodal, convergent, and divergent steps

	Step Visits^1^	Steps Preceding	Steps Following
**Nodal Step**			
Descending & sigmoid colon mobilization (medial-to-lateral)	86	13	11
Descending & sigmoid colon mobilization (lateral-to-medial)	87	16	15
**Convergent Step**			
Rectal mobilization (medial-to-lateral)	92	13	7
**Divergent Step**			
Rectal transection	39	6	12

1 Cumulative visits during 31 proctectomies
